# To connect between worlds, to bridge over gaps: learning about the complex role of cultural mediators in perinatal health promotion from a case in Israel

**DOI:** 10.1186/s12939-020-01161-z

**Published:** 2020-04-25

**Authors:** Hagit Peres, Rachel Sharaby

**Affiliations:** Ashkelon Academic College, Har Amasa, D.N. Drom Har Hebron, 9040300 Ashkelon, Israel

**Keywords:** Mediators, Cultural competence, Health promotion, Social determinants of health, Perinatal health

## Abstract

**Background:**

Reduction of health gaps between ethno-cultural groups has become a major concern for health services, with a strong emphasis on eliminating social and cultural barriers and improving accessibility for diverse populations.

**Methods:**

The study is based on a Participatory Action Research where an involved researcher accompanied the project for a decade, as well as on eleven in-depth interviews with Bedouin women-mediators working in a perinatal health promotion project in Israel.

**Results:**

The research analyzes the work of Bedouin women health mediators who mediate between their Bedouin community and institutional health services and bridge over cultural gaps. The study presents the complex task of transferring messages across cultures, dealing with socio-cultural imperatives and the intricacy of multilayered power relations. The findings reveal an evolving process, beginning with a pragmatic mediation model in which the mediators are limited to instruction of pre-defined health materials, toward a transformative model of creating a ground for encouraging the mediators to act creatively according to socio-cultural circumstances.

**Conclusion:**

The research elaborates on the adoption and implementation of the transformative approach in mediation and provides further understanding of the complexity of mediation role in sensitive issues such as pregnancy, birth and infant care.

## Introduction

The need to bridge over gaps in health between populations has occupied health institutions around the world for several decades. The common basic assumption in the past was that sickness can be cured solely with bio-medical knowledge. This has gradually been replaced by the understanding that social and cultural factors that define health should also be considered. Reduction of health gaps between ethno-cultural groups has become a major goal, with the aim of leading to a significant and fundamental improvement in services and in their accessibility to diverse populations [1, 2].

In the early twenty-first century, the World Health Organization (WHO) set a goal of “closing health gaps within one generation”, adopting an innovative perception regarding factors causing health and disease and adjusted modes of action. The position of the WHO appears in a series of documents of the Commission of the Social Determinants of Health (CSDH). The new holistic viewpoint takes diverse factors that affect health into account: clean water, proper nutrition, access to health services, education, work, leisure, housing and a supportive social-cultural environment [3, 4]. The committee claims that health gaps are related to unequal division of power, income and assets, which are translated into non-egalitarian policies and programs [5].

The consensus that socioeconomic status is crucial in dictating people’s health led to the identification of cultural barriers and socioeconomic limitations that create difficulties, mainly among disenfranchised populations. Women from minority groups are particularly vulnerable. Lack of education, low income and low social status harm their ability to maintain good health [6, 7].

The social-cultural knowledge that supplies explanations for the strong connection between high morbidity and inequality is not new in the social sciences. There is no doubt that health measures are better in countries with good governance, low unemployment, high standard of living and economic stability. However, great gaps between statuses, genders and ethnic groups exist even in rich countries [7, 8]. This trend necessitates broad application of sociological and anthropological knowledge when constructing and managing health promotion programs [9].

The call to remove cultural barriers has grown in the last decade, in light of the West’s coping with global immigration waves and caring for ethnic minorities [10]. The relationship between health and culture led to the establishment of the Care Quality Commission by the National Health Services in Britain, headed by the anthropologist David Napier. This commission outlined a renewed perception of health-promoting policies, which places culture at the center of healthcare, claiming that culture plays a crucial role in understanding how people perceive and maintain their health. The commission’s conclusions were published in *The Lancet* and claimed that understanding and assimilating culture and its implications are crucial in health-oriented actions. The commission recommended identification of new models of quality of life and treatment, and their implementation among cultural groups [11].

## Cultural competence as a measure for the quality of medicine

Cultural competence was originally defined as a set of congruent behaviors, attitudes and policies which come together in a system, agency or among professionals, that enables effective work in cross-cultural situations [12]. The definition is based on the consensus that culture mediates between the ways in which people understand and manage treatments and relations with health institutions, their expectations from the treatment and health literacy [13].

Later definitions attribute self-awareness, cultural knowledge, acceptance of cultural differences, and skills adapted for treating “others” to the cultural competence of caregivers [14]. The construct of cultural competence is perceived as an operative tool for reducing gaps in health, connecting between the quality and efficacy of healthcare and the aspiration to provide equal care [15]. Implementation of culturally competent and compatible health services dictates new policy strategies and standards for health services around the world [16, 17].

The process of the institutionalization and integration of cultural competence as a leading approach in health institutions includes several milestones. The pioneering National Standards for Culturally and Linguistically Appropriate Services (CLAS) program affords operational standards of culturally competent health services that are still used in health services today [18, 19]. A network of Migrant-Friendly Hospitals (MFH) was established in the European Union in 2002 [20] with the aim of establishing an inclusive perception and practical tools for supplying health services to populations from different cultural backgrounds [21, 22].

Culture-guided programs in additional countries, including Australia, Canada, Britain and New Zealand, referred to different aspects of cultural competence. The United States emphasized the need for setting standards, Australia encouraged cooperation and dignity for communities, and England focused on a culturally diverse workforce. Canada, Australia and New Zealand stressed the quality of the therapeutic interaction via the concept “cultural safety”, which puts the patient in the center and espouses identification of cultural elements that endanger the treatment if they are not taken into account [23–25].

The construct cultural competence was also criticized as a concept that may encourage conceptual superficiality and stereotypification. In many places, a tendency to find only technical and administrative solutions for linguistic and cultural problems is apparent, instead of coping with deep meanings of cultural difference [11, 26, 27]. Napier and the commission headed by him indicated the danger embedded in expanding essentialization and stereotypification via the term culture and called for constructing a therapeutic infrastructure that is attentive to the patient, encourages patients to make independent and rational choices and ensures space for the expression of preferences as well as concerns. This is a call for renewed thinking about cultural competence that develops the ability to build trust and conduct negotiation regarding the therapeutic process, out of attention to the patients’ experiences [11].

## Policy of inclusion: cultural competence in Israel

Israel, which is coping with cultural and linguistic differences and prolonged waves of immigration, has somewhat belatedly joined the global trend of developing cultural competence in the health services. Local studies that indicate gaps and inequality in health [19, 28] are motivating policy makers to create a new agenda that meets the standards of Western countries. In addition to the need for decentralization of infrastructures and resources (distribution of doctors, medical services, number of hospital beds, etc.), it is apparent that cultural barriers that influence the consumption of health services must be bridged [29].

In 2011, the Ministry of Health published a Director-General Directive (7/11) that stated that the health system’s needs to cope with basic needs of culturally diverse populations is based on humanistic values, as well as on legal aspects related to the definition of a reasonable and appropriate standard of care. This was the first time that cultural diversity was recognized as an issue that demands consideration in the medical care framework in Israel. The directive presented an outline for implementation of cultural competence in the health system as a tool for reducing health gaps. However, the directive was not backed by specific legislation and budgets, which led to difficulties in its full and obligatory implementation by health services.

Follow-up documents expand reference to implementation of cultural competence in health services [29]. Institutional persons-in-charge were appointed and trained for promotion of cultural accessibility, including medical translation services, and medical mediators and interpreters were hired by some institutions. Several non-government agencies act for training medical staff in the development of cultural competence and linguistic accessibility [30].

In 2015, the Ministry of Health published another internal document that expands the role definition and activity of cultural mediators in the health services, as follows: The role of mediator is not limited to medical translation, but rather includes bridging over cultural gaps, including perceptions, beliefs, worlds of knowledge, between the staff and patients from unique target groups … The mediator’s activity will contribute to strengthening the trust and mutual understanding between the service receivers and providers, will reduce tensions and difficulties that originate in language and culture differences, and will lead to a more adapted, high quality and effective treatment and service process for both the patients and the service providers.^1^ This document indicates recognition of the importance of the health mediators’ role. However, it does not include allocation of resources and positions and its application is therefore limited.

## Intercultural mediation

Since the 1970s, and in particular since the 1990s, mediation (alternative dispute resolution) has become common around the world and in Israel. It has become institutionalized as an alternative legal procedure for dispute resolutions, or as a service to the community, that is applied in diverse social and legal contexts in different fields [31, 32]. Mediation helps consolidate mutual agreements that lead to a joint solution which is acceptable to the parties and to mutual recognition. In essence, mediation represents a worldview where each of the mediated parties gains from the conflictual situation [33].

The common model in mediation practice is called the pragmatic model, which was founded by Fisher and Uri [31, 33]. This model focuses on interests that motivate the conflict and not on attitudes, and its goal is to end the dispute with an immediate agreement. The mediator focuses on the solvable consensus issues, avoids controversial areas and motivates the parties to reach an agreement. The conflict perception of this approach is based on the assumption that the person’s desires stem from his needs and interests. Conflict is created when a non-realized expectation arises.

Bush and Folger’s transformative model [31, 34] presents an alternative critical theoretical model. Transformative mediation does not strive to an immediate end of the dispute, but rather to a change in the attitude of the disputing parties. The model’s goals are to strengthen the parties’ communication skills and develop empowerment and mutual recognition abilities. Transformative mediation perceives the dispute as a positive construct. It suggests referring to the dispute not as a problem, but as an opportunity for growth in two critical human dimensions: strengthening of the self and recognition of the other. The mediation process therefore often ends not only in resolution of the dispute, but also in a renewal of the relations between the parties based on mutual trust, which was lost during the dispute.

The meaning of mediation in cultural conflicts is a “celebration of the difference” and cessation of its denial [35]. The intercultural mediator must understand the values, behaviors, feelings and needs of both parties. In this context, intercultural mediation is responsible for creating a connection between different languages and cultures, such as: values, norms, practices, etc. [36]. These and other scholars also compared the intercultural mediation action to the translation work, which is an interpretive act that requires linguistic and cultural knowledge that enables a person outside of the culture to understand something within that culture. In addition to being a translator between cultures, he/she also functions as a balancer of contrasts between them and helps preserve their unique identity [37, 38].

Salacuse [39] suggested several ways for bridging over a cultural gap: to learn about the culture of the “other”, to abstain from referring to stereotypic perceptions, to help the “other” become more familiar, and to include elements from both cultures [40]. McLeod compared the work of the intercultural mediator to that of a teacher, since similarly to the teacher, the mediator needs good communication skills, broad knowledge on cultures and needs to use it in order to teach people from one culture about the other.

Intercultural mediators play a crucial role in organizations. Their main role is to mediate between cultures that represent different ways and perceptions for achieving the same goal, which is focused on advancing personal or social wellbeing [41]. Intercultural mediation focuses on the relations between service providers and minority groups with the aim of finding a middle way in spite of and out of the cultural differences. The mediators therefore focus on clarifying the verbal and cultural messages, while finding the common elements. This role includes using mediation, coordination, diagnosis, consultation, explanation, support, organization and empowerment skills [42].

Intercultural mediation is also perceived as a “cultural sensitivity skill”, whose uniqueness is the ability to enable effective, dignified and adapted meeting between different cultures. In this context, the intercultural mediator must often influence the mediation process and employ skills of defending the rights of members of the minority culture [41]. It appears that the influence of the intercultural mediator stems from the degree of hope and confidence that he/she arouses by his/her actual presence in the organization, and in the personal model that he/she represents, between the culture of origin and the new culture [42].

Thus, intercultural mediation combines a worldview, skills and knowledge which can train an organization for intercultural work. The presence of an intercultural mediator in the organization may facilitate the coping with intercultural barriers. Furthermore, when the intercultural mediator acts in multi-professional and multicultural teams, he/she can supply cultural viewpoints and suggest solutions that express the cultural diversity [43].

Public recognition of intercultural mediators has dual significance: direct admission of the necessity of the role and social willingness to accept the ideological ideas at the basis of the role, which means intercultural integration out of a formal recognition for the place and expression of different cultures in society [41].

In this article we discuss the adoption and implementation of a transformative approach in mediation in the work of women mediators in the Bedouin society.

## The program for promotion of health in Bedouin society

At the end of 2017, the Bedouin population in southern Israel included 250 thousand people [44]. About two-thirds live in permanent towns and the rest live in unrecognized villages, without any municipal infrastructures. This is originally a tribal society, that until the establishment of the State of Israel in 1948 subsisted on small farms and growing sheep. Upon the establishment of the state, the Bedouin population was forced to make drastic changes in its lifestyle, which included a drastic disrupture of the original tribal structure, and a significant change in way of living.

The state encouraged settlement of the Bedouin population in permanent settlements that were gradually built. This obligated a drastic cultural transition to modernization [45]. The state did not have the sensitivity of accompanying the transition to a market economy by supplying the necessary tools and resources. This process left the adults helpless in coping with the changing world and posed difficulties in inclusion of the youths in the modern world. The Bedouin settlements in the Negev are found in the lowest socioeconomic cluster [46]. As a result, their health situation is poor compared to the Jewish population in southern Israel [47, 48].

Women, who in the traditional family fulfilled a significant productive role, lost their economic occupations in the sedentarization process, and became absolutely dependent on the men. The urbanization process prevented free movement within the new public domain for many of them. To date, many are dependent on the accompaniment of a man for going into the public domain, including for medical treatment. Young women who aspire to an education and find a job have difficulty in breaking the family and social bonds [49, 50]. Traditionally, women still marry men from their extended family (with preference for paternal cousins). This custom increases the medical risk for genetic defects, which are a dominant factor for infant mortality in Bedouin society [51, 52].

Infant mortality is universally influenced by many socio-demographic variables [53]. The infant mortality rate in Bedouin society is significantly higher compared to the general population in Israel [51].

The high mortality and morbidity among Bedouin children were the main motive for establishing the “Program for Reducing Infant Mortality in Bedouin Society”. The program was implemented between 1995 and 2015 by initiative of the Ministry of Health and the Department of Epidemiology of the Faculty of Health Sciences at Ben Gurion University in the Negev, with collaboration of the relevant health services. The project was managed by the academic institute and was headed by Prof. Ilana Shoham-Vardi.

The project employed 10–15 women mediators who worked in the Mother and Child clinics (MCH) that serve the Bedouin society. The project goals included: increasing knowledge and awareness of risks for the fetus during pregnancy and for infants after birth; reducing the risk of giving birth to babies with congenital malformations by adoption of recommended behavior patterns; improving utilization of health services for women of fertility age and infants (Annual Report of the Project, 2009).

Initially, the main role designed for the Bedouin mediators was “to provide instructive materials” to women who visited the MCH clinics in the local Arabic dialect. During the first ten years, the instructors’ training programs therefore focused on instructional subjects such as the use of folic acid for reducing neural tube birth defects, genetic and congenital diseases, preterm tests during pregnancy, etc. During the second decade of the program, greater focus was placed on the social and cultural conflictual situations experienced by the mediators, and the mediation role was expanded to “bridging language and cultural barriers” between the Hebrew-speaking medical team and the Arabic-speaking Bedouin women who visited to the clinics. For example, in 2012 the mediators were provided with a medical translation course, which beside linguistic techniques for translation emphasized cultural mediation. The course also included a training section with the MCH nurses. During the last years, the in-practice training focused on role playing, where conflictual socio-cultural situations were raised and discussed with emphasis on protecting and confining the mediator’s role to providing the information, but not to advising pregnant women in difficult decisions about their pregnancies. As will be shown later, the project has evolved, since the mediators’ work turned out to be much more complex than just a delivery of instructive materials.

## The research goal and methodology

This study examines the way in which the mediators, as women belonging to the Bedouin community, transmit the health messages of the medical establishment. We analyze how women-mediators who come from a similar cultural background to that of the women patients, and are recruited and employed by the establishment, cope with the complex role of mediating cultural gaps. A deeper understanding of the complex challenges facing the mediators is essential for optimal implementation of the mediation program, in particular regarding sensitive issues such as pregnancy and birth. This will enable us to understand the adoption and assimilation of a transformative approach in mediation.

Qualitative research investigates social phenomena in natural settings, with an attempt to produce meaning or interpretation of an effect which the participants bring with them [54]. Data collection via an in-depth interview enables expression of the insights and experiences of the interviewee, using her concepts and point of view. This approach is espoused by feminist researchers as a mean that enables access to opinions, thoughts and experiences, with the aim of sounding the women’s voice [55, 56].

Qualitative research has prominent advantages in cross-culture contexts. The direct connection between the researcher and the participants enables reducing the inherent mistrust that exists in relationships that cross ethnic and status groups [57].

The present article is based on a Participatory Action Research (PAR) of one of the authors, who accompanied the project from 2005 to 2014. The author’s accompaniment included taking an active part in recruiting and training the mediators in teamwork with a supervising nurse of the Ministry of Health. Our analysis focuses on in-depth interviews held with 11 mediators who worked at that time. In order to describe the evolution of the project, we complemented the interviews with data obtained through ten years of involvement in the project management (such as annual reports, reports from steering committee meetings, and training sessions for the mediators co-conducted by one of the authors with the Ministry of health’s nurse in charge of the project.

In the spirit of the works of Paulo Friere [58], the main aim of such a study is “to lead to a more just society via activity for transformative change” [56] p.188. The prolonged acquaintance enabled the researcher to accompany the development processes in the lives and work of the mediators in a supportive and relaxed atmosphere. Nonetheless, the gaps in the power relations between the author who belongs to the employing and operating side as well as to the cultural hegemony and the scientific authority did not disappear completely and are also present in the interviews that were held toward the end of the program.

The mediators were recruited for the role based on job interviews and participated in several weeks of intensive preliminary training. Bedouin women who had 12 years of education and good mastery of Hebrew were accepted for this job, with preference for women who were mothers. The following table presents detailed characteristics of the interviewees at the end of 2014.

**Table:** 

Name (fictitious)	Age	Family status	Working days and # of clinics	Location of clinic	Seniority in the project
Mariam	30–40	Married + 2	2 (5)	Bedouin Town	8 years^a^
Manal	30–40	Married + 5	1 (3)	Unrecognized village	5 years^a^
Alia	~ 30	Married + 5	1 (2)	Village in process of recognition	Left after 2 years
Salsabil	~ 35	Married + 3	2 (4)	Town serving the Bedouin population	6 years^a^
Rola	20–30	Single	2 (4)	Bedouin town	5 years
Haula	~ 30	Married + 6	2 (5)	Hospital maternity ward	Veteran, 15 years
Hiam	40–50	Married + 10 grandmother	3 (5)	Bedouin town	Veteran, 15 years
Inas	20–25	Married + 2	1 (5)	Bedouin town	3 years
Fairuz	30–40	Married + 3	1 (4)	Bedouin town	7 years
Suaad	40–50	Married + 9 grandmother	1 (3)	Bedouin town	8 years^a^
Nirmin	40–50	Married + 1	2 (5)	A city that serves the Bedouin population	3 years

^a^Not consecutively

The mediators worked in MCH clinics, during hours convenient for mothers. They received a minimal wage for their work, and sometimes, due to lack of a budget, their work hours were cut. Many families restricted the mediators to work only to certain clinics, and often asked them to stop working for various reasons.

The interviews with the mediators took place individually, in the clinics, the project’s offices, or in some other place according to their choice. After transcribing the recordings, thematic content analysis was performed. This method enables focusing on the individual and her world of meaning, with the aim of exposing processes and experiences that take place in a broad social-cultural context [59].

## Findings

### “To be in the middle”: the mediators’ role perception

All mediators indicated how the role positions them “in the middle”. The position in the middle causes the mediation role to be complex on several levels: between the medical system and the women who visit the clinic; between traditional Bedouin society and modern society; between the aspiration to independence and freedom and the connection to values of a traditional and patriarchic society; and between the woman and her pregnancy and fetus. Inas explained that “the middle”, in all its different dimensions, arouses contradictory feelings, satisfaction and esteem, but also criticism. Fairuz stressed the tension between the different expectations from her by the medical team and her community.

**Inas**: *I saw that the mediation is the connection between me and the woman, and between the woman and the nurse, with me in the middle. I also saw myself between the tests and the things that are important to the woman and her baby. I saw myself intervening between the woman and her fetus … It is as if I grab the rope to where it wants to go, help her walk if she cannot walk. I help her in how to get up and how to sit down. … Most of the time, the special place of being in the middle is a comfortable place. [but] I sometimes see myself in the wrong place because I interfere in something personal. I see that the baby and the woman’s tests are something personal for her or between her and her baby, and I interfere, and sometimes this is good and sometimes it is bad.*

**Fairuz**: *I am a mediator. Mediator for me is not a simple thing. It is real. It is next to the nurse. This is one person who continues the other. Also in our village, my workplace as a mediator is something important for us.*

### To take the load off their heart and their back

The mediation role creates an opportunity for a personal and open encounter between the mediator and pregnant women and mothers from different extended families which they would not encounter under other circumstances. The feminine space at the MCH clinics provides the women an opportunity to share common experiences of pregnancy and motherhood with other “peer” women. The mediators indicated the importance and intensity of such personal and intimate encounters. The principle of confidentiality to which the mediators are committed as part of their professional code is what, according to them, enables them to gain “other” women’s trust. Women who were very reluctant to share personal problems with their relatives were very happy to find a confidential “stranger” in the mediator with whom they could “unload their burden from their backs”. The mediators expressed satisfaction from the women’s demonstrations of confidence. However, crossing the boundaries into the personal and confidential was burdensome for them, with no evidence for any professional emotional support to the mediators:

**Suaad**: *Some women want to talk with me about other topics, after the end of the instruction. They are comfortable talking with a person who is not from their family, and unload everything they have. They tell what they have and unburden the load from their hearts and backs. They say that they do not want anyone to know what they told me. For me, this is as if they spoke with themselves. And there are such things that I hear and take home, and this has a psychological effect on me.*

**Hiam**: *I never talked about a woman who has such and such. They knew that I maintain confidentiality, and because of this, they may have loved me more. Because there are things that they do not want anyone to know about. I do not only work, I am like the women’s psychologist. I sit with them, make them laugh, give them the feeling that I am their friend.*

### Knowledge is power

The mediators describe their work as rewarding and satisfying because it makes a significant difference in the women’s experience of visiting the MCH. They report on a change in the women’s approach toward using health services over time, and indicate the knowledge which they pass into the hands of the women as a source of power.

**Suaad**: *This work is very important. When I began eight years ago, the women did not have such information. Today they also read and surf the internet and accept my instructions better than at the beginning.*

**Nirmin**: *First of all, the knowledge they receive, know what this is and that is, and their rights and what [prenatal] tests they should have. They also turn to the nurses more often. At the beginning of work, they would not knock on the door and complain about waiting a long time. Or ask what this or that test does. Today they have more knowledge and [they] are stronger. This is a result of our instruction, it indicates how important our instruction is. I have responsibility toward the women. It gives them awareness and I think that I strengthen them. Knowledge is like giving a person a weapon so that he can protect himself. Knowledge is power.*

### “I am one of them”

The mediators are careful to not describe themselves as having higher authority and status than the women visiting the clinics. Rather, they emphasize their closeness to the women, the fact that they are part of the community and the community’s appreciation of their role, in spite of the training and role which distinguish them.

**Nirmin**: *I am one of them, and would not like to be treated [otherwise]. Socially, this is great. The people are happy to know and speak and share. To ask for help. People are not afraid and do not keep their distance from me.*

**Haula**: *I am not a doctor. I am a human being. Sometimes I tell the women that “you also have knowledge, and I also learn from you”. It’s not as if I am the boss and go down to their level. They need this. When you speak down to the woman, she will not accept you. I am not better than she is. There may be things that she knows and I do not.*

### Coping with medical instructions and messages

Decreasing the number of congenital birth defects is a charged issue. In a society where marriages are arranged, testing for mutations for genetic diseases may significantly harm the chances of women who are carriers of genetic mutations to marry [60]. The medical establishment encourages women to undergo prenatal testing, but aborting the pregnancy arouses significant opposition in Islam. Many Bedouin women avoid prenatal tests so as not to cope with this dilemma [61]. The mediators are instructed to transmit messages that may contradict their religion:

**Rola**: *I give instruction, but I myself cannot put the women into this bubble [prenatal tests and coping with their results] and force them to undergo amniocentesis. I don’t know. The test is also dangerous. If something bad will happen to them, it will be my responsibility. Therefore, I do not take this responsibility.*

The system has pragmatic expectations from the mediators: explicit recommendation to perform prenatal tests and explaining the increased risk for hereditary diseases in consanguineous marriages, and indirectly to recommend avoiding such marriages, contrary to the traditional norm. An illustration of this conflict was expressed by a mediator who herself avoided tests which she is instructed to recommend:

**Inas**: *[In my first pregnancy] I felt calm even though in the first test, the fetal protein, was abnormal. I said: What will I do? I knew that there was a chance that the results will be abnormal and I also say this to the women, that I also had such-and-such a result … “Do all the tests and then decide whether it is good or bad”.*

In order to solve the dissonance, Inas shares her test results with the women, but does not reveal her personal decision of not doing additional tests, as recommended. Rather, she suggests that the women decide for themselves.

### Creative instruction

In the first years, the mediators were instructed to read pre-written instructions verbatim and in full. These instructions reflect a pragmatic and passive mediation perception, that does not believe in the mediators’ ability to exercise discretion. This type of instruction led to disinterest and impatience by the women, and the mediators were therefore instructed to exercise more discretion and adapt the content and duration of the instruction. This change enabled the mediators to be active and creative in their instruction:

**Inas**: *I read all the instructional kits several times at home and learned them, and shortened them in my head to prepare for communicating them. In the instructional kits on Genetics and Hereditary Diseases, for example, I saw that there are things that are repeated. I shorten. I know which are the important and interesting pages and those I say. If I would read each page I would not have time to finish, and the woman would be bored and would leave. I begin from the pregnancy stage in which the woman is found and begin with these tests.*

As the project evolved, and the leading team received feedbacks from the field, adaptations were made that gave more freedom to the mediators to respond to challenging situations. Most mediators had accumulated the required experience and self-confidence to share their criticism of the instructional materials with the leading team, and acquired the necessary instructive skills to conduct their role as mediators in a much more acceptable mode.

### Conflict between cultural codes

Working in public spheres such as MCHs pose some conflictual situations for the more traditional Bedouin women: the imperative of avoiding meeting with non-familiar men, having to show their faces and talking directly to men. Most of the mediators wear traditional clothes and cover their heads with a scarf that reveals their faces. At their request, the project bought white coats for them, similarly to those worn by the nurses. The coats comprise a symbol of their professional status as mediators and impart them legitimization to conduct talks with non-familial men, a behavior that is strictly forbidden and violently punished within some Bedouin families, but which is obviously necessary within the medical context of the clinics. Some families insist that their women wear the *hijab* (a cloth that covers most of their face), especially when strange men are nearby. However, the nurses ask the women who visit the clinics, and certainly the mediators, to remove the *hijab* from covering their faces during the clinical encounters, as well as during the mediators’ instruction.

The mediator **Manal** found a creative solution to the conflicting demands between the social requirement from some women (mediators as well as patients) to wear a hijab in the public sphere and the professional necessity to uncover their faces while in a medical encounter:

*When a woman [with hijab] enters [the clinic], her face is like this (demonstrates facial cover by the hijab). The nurse asks that she take it off, and some do not agree to do so. [In order to avoid an angry reaction from the nurse], I said, all right, take it off. You came to the nurse and the nurse does not accept you like this. The nurse then told her, how beautiful you are, and the woman was pleased. At first I [myself] always took off my hijab [but only while] in my room. It bothers me to speak with a woman with my face covered. I tell the woman, take off the cover too, we are women and the door is closed.*

### An opportunity for change in the community

The work as mediators confronts them with the social problems in Bedouin society: high rates of congenital birth defects, as well as the difficult family and social situations of families having children with congenital defects, and particularly meeting women with difficult pregnancies. Some of the mediators identify the visits at the clinics as an opportunity for creating change and preventing the suffering involved in raising children with serious defects by performing prenatal tests and performing pregnancy terminations wherever grave congenital defects are detected.

**Rola**: *First of all, I feel sorry for the woman. She is still very young, and could have terminated the pregnancy. From now on she will live her life with a handicapped child and that is difficult. It is true that God created him, but the responsibility for the tests and [for] his [future] care falls on people.*

Rola presents her compassion, especially for the young mothers who have children with congenital defects. Having experience in her own family, she advocates taking the responsibility and agency as the people who will cope with the burden of caring for a handicapped child, and not to leave it to God. However, this approach is widely unaccepted by many Bedouin people. Another extremely sensitive issue which the mediators approach with caution is consanguineous marriages:

**Salsabil**: *Regarding consanguineous marriage, I don’t talk to them about this. I do not talk about the diseases related to this during the instruction, but rather I call these hereditary diseases. However, I talk about this later during the instruction, because it is very important. Because if I say “consanguineous marriage” at the beginning, they will not come … First of all, she says that she is already married to her cousin 10 years and they have four children and thank God they are all healthy. But I recommend and tell them, that if someone wants to marry … you can see that the Bedouin are beginning to marry outside the family. I explain the importance and problem of consanguineous marriage. But if you do want to marry in the family or a cousin, then this should be with tests, and make sure tests are performed [to check the fetus at pregnancy].*

In Bedouin society, similarly to other traditional societies, the attitude toward people with disabilities is ambivalent. On the one hand, Islam views them as products of Divine creation, and forbids the termination of pregnancies after 120 days. On the other hand, there is social rejection of disabled children and their families. The mediators described their attempts to support the mothers:

**Hiam**: *A woman who brought a child with Down syndrome to the clinic. When she came to the clinic with the child for the first time, she cried a lot. I took her to a room and we sat together, and I told her that thank God her case is not severe. Some women see such a child and look down on the mother and the child. I do not. I see her as my own child. I hug her and tell her how beautiful she is and how nicely she is dressed, and she does not feel that she has a sick child. This is where I can help people with problems. Any woman could have such a child. So what? It is not shameful.*

Exposure to women who suffer from domestic violence creates a dilemma for the mediators: should they pass this information to the nurse and involve the welfare services, or uphold their promise to the women to maintain confidentiality:

**Alia**: *Some women have bruises on their faces from being hit by their husband. One day a woman came with marks on her face. She looked at me and said, this is from my husband. I immediately told this to the nurse. That same day we made a home visit and saw that the situation is not good. The husband saw that we took an interest in his wife and came for a home visit and stopped hitting her. She told me, “from the day that you told the nurse, and came to visit me, my life changed. Thank you.”*

The mediation work exposes a difficult social picture toward women, but also imparts a perspective of change and a sense of involvement in creating it:

**Fairuz**: *The Bedouin society of 14 years ago is not as it is today. Once I would have been afraid to be in a place where there is a man and talk with his wife, that he would perhaps say what he wants of her. Today things are different, and I can also argue with a man, because this gives confidence and strengthens the women. At first everything was difficult for Bedouin women. The husbands brought them and waited by the door. Today I do not see this. My message is that all Bedouin woman should go out and learn.*

**Mariam**: *I think that in Bedouin society women are the weakest body. Women were given much information, but men were not. And this is a problem. We strengthen the weak party and the other party is dominant and everything is in his hands. I feel that I give the women tools, that they will change their lives, and also the future of the coming generations. That this generation will not suffer as the previous generation suffered. I am optimistic and love to help and change things, very slowly. Patience is necessary, and slowly things will change.*

## Discussion and conclusions

The last decades were characterized by a search for successful models of assimilation of social competence and sensitivity in health services in many countries. The work of mediation and the employment of intercultural mediators create an opportunity for the emergence of meaningful dialogue between medical teams and excluded minority groups.

This article explores a perceptual and operative change in a mediation project, which pertains to the meaning of transformative mediation and the manner in which it evolves. We suggest that transformational mediation makes a substantive contribution to promotion of social empowerment that eventually reduces health inequalities. The insights from the case of the Bedouin mediators is, in our opinion, an example for other mediation frameworks, in particular in projects among marginalized communities.

The initial goal of the program discussed in the present article was pragmatic: dissemination of medically created information for reduction of infant mortality, and the mediators were employed in order to communicate instructions to Bedouin pregnant women and mothers.

With time, transformative signs began to appear within the project. Some were encouraged by the project leaders, and others grew among the mediators out of needs dictated by the field. Separation from the model that dictates structured cultural enforcement (top-down) occurred gradually, and a transformative model that encourages negotiation, dialogue, mutual learning and creation of a fertile intercultural space has developed, in which needs, new viewpoints and preferences of the population are raised. This was a gradual process, and the pragmatic model did not completely disappear. Rather, the transformative model contributed depth, meaning and relevance to the lives of the patients during medical encounters.

Bischoff et al.’s literature review [62] on the role definitions of medical interpreters indicates a broad range of roles that they perform. In addition to verbal translation, the role includes a growing number of tasks, inter alia intercultural mediation, advocacy and representation of the patient’s voice, administrative help, etc. The holistic range of tasks described by Bischoff is compatible with the initial process we described in this article, since with time, the roles of the mediators expand beyond the communication of medical information, and raise viewpoints and modes of treatment that come into being within the cultural, social and political contexts.

Most of the interviewed health mediators worked in the project for years. They experience the perceptual changes and indicate that they themselves underwent changes. It is evident from their testimonies that the transformative model expands their possibilities of acting vis-à-vis the women patients, enables them empowering activity within a more significant space, and is therefore empowering and rewarding for them. Nonetheless, some still have difficulties implementing independent considerations, and prefer to adhere to the texts dictated by the medical-scientific authority. Adherence to an authority comprises a certain solution when conflicts and ethical social-cultural conflicts arise.

Covert and quiet resistance of the mediators to the messages transmitted by the medical system were observed in some of the interviews. This is a minor form of resistance that avoids overt and direct confrontation with authority in power, as described by De Certeau [63]. Even when the mediators feel uncomfortable with messages that contradict their tradition, they avoid open confrontation and do not relinquish the opportunity that the institution afforded them to participate in the complex medical arena and contribute to the community wherever they can.

Thus, some of the mediators who are asked to communicate a message that calls on the women to perform prenatal tests, avoid these tests themselves, out of an understanding that the test results may pose a dilemma of terminating a compromised pregnancy. The mediators comply with their task and convey the required information and encourage the women to make an independent and informed decision. Instructive workshops held for the mediators included many discussions on the importance of non-directive instruction, avoiding responsibility for their decisions. The understanding that women are able to make educated and independent decisions is transformative and generates change.

Transformative mediation requires knowledge and instruction skills. In line with Napiers’ commission, transformative mediation may be an appropriate approach that aims to avoid essentialization and stereotypification by careful listening to the voices in the field without prejudgment. The mediators emphasize feelings of empathy and identification with women in sensitive situations, which necessitates curbing their emotional involvement toward them. They learn to contain decisions that are “external” to them, without judging the women and avoid giving advice. Complex skills and concepts, such as non-directive guidance, are acquired gradually and require experience and emotional maturity. The transformative model creates internal motivation that stems from an empowering mission.

The mediators are well-acquainted with poverty, unemployment and social marginality in many Bedouin families, and are also witnesses to the privileged life of those who belong to the social hegemony. Their role places a social mirror in front of them, that on the one hand arouses helplessness, and on the other hand a strong desire to act for a change and to be part of it. As such, the mediation role requires resources, support and great mental strength [64].

The mediators’ position is in the “middle” in many ways. The middle is a connecting place and a builder of bridges, but also a place laden with conflicts that leaves a bitter taste.

An empowering, protective, supportive, affording knowledge, advocating, sometimes hugging and non-judgmental experience develops alongside the experience of difficulty and weakness. The mediators comprise a source of inspiration and role model for the women, but are themselves sometimes weakened. Sometimes they succeed in communicating change-generating messages, which the employers did not originally intend. Via the mediation they begin to think, in depth, about existential issues such as what is a life worth living, how women/mothers should be treated within their society, especially those caring for babies with defects that their society forbids aborting; about relationships between mediators and the medical establishment; and ultimately, their moral relationships with God.

In our opinion, pragmatic elements of communication of medical information, that conceals an expectation for increased compliance with recommendations of the authoritative body which are at the basis of the medical treatment [65], will not disappear completely. However, transformative elements that impart depth and meaning and enable construction of a fertile, containing and more egalitarian relationship can exist alongside it. Our paper is confined in its focus to the mediators and does not cover processes of change among the medical team at the clinics. In spite of hints provided by the mediators that point to an evolution in their relationships with some of the nurses, we could not make any systematic observation about a major change in the medical team’s approach toward the mediators nor the Bedouin patients.

Most of the mediators gain a sense of belonging and esteem by the medical team. Such inclusion is socially rewarding, but also emphasizes the dissonance between the sense of emotional belonging on the one hand, and the institutional exclusion as expressed by their poor employment conditions on the other hand. Thus, the more effort that the mediators invest in their mediation role, the stronger is their resentment of their low position in the hierarchy and the insultingly low material reward.

Employment of mediators from excluded communities has advantages, but also poses challenges. The position of the mediators exposes them to criticism from their own community, but also sets expectations and demands from the medical system, which the mediators have difficulty implementing. Heads of intercultural mediation programs, in particular in disenfranchised communities, therefore need to understand that the mediators are themselves a vulnerable population that needs emotional as well as social support, social sensitivity, protection and empowerment, in addition to provision of informative knowledge, instructional skills and awareness of health situations, so that they will be able to contribute to their target population.

## Data Availability

Data for this study include documented reports and the researchers’ reflections collected over 10 years, as well as transcription of eleven audio-recorded interviews with the mediators (in Hebrew). All the collected material was processed in Hebrew, while some of the training sessions with the mediators were held in Arabic and documented in Hebrew. Data will be available upon request of the editorial board, with the condition of protecting the identity of the interviewees upon commitment to protect their privacy.

## Abbreviations

WHO: World Health Organization
CSDH: Commission of the Social Determinants of Health
CLAS: Culturally and Linguistically Appropriate Services
MFH: Migrant-Friendly Hospitals
MCH: Mother and Child clinics
PAR: Participatory Action Research

## Footnotes

1 One of the authors served as a consultant to the Ministry of Health on role definitions of mediators in the health domain.
